# Current Trends in Micro and Nano Manufacturing

**DOI:** 10.3390/mi13122058

**Published:** 2022-11-24

**Authors:** Joško Valentinčič

**Affiliations:** Faculty of Mechanical Engineering, University of Ljubljana, Aškerčeva 6, 1000 Ljubljana, Slovenia; jv@fs.uni-lj.si

Micro and nano manufacturing technologies can be used to machine materials ranging from polymers and metals to ceramics and other modern high-performance materials. Some of these technologies enable cost-effective mass production; others are effectively used to produce tools for high-volume production. Hence, they are complementary to micro system technologies used to produce micro electromechanical systems (MEMSs).

The World Congress on Micro and Nano manufacturing (WCMNM) is a concluding step in establishing a global forum of academics and industrial practitioners involved in micro manufacturing technologies, micro part assembly and handling, surface engineering, micro factories, micro sensors and systems, and nanotechnologies. It is a joint event of three organisations: the 4M Association (Europe), the International Institution for Micro Manufacturing (Americas) and the International Forum on Micro Manufacturing (Asia). The first Multi-Material Micro Manufacture (4M) conference dedicated to micro manufacturing and micro assembly was held in 2005 at the Forschungszentrum Karlsruhe. It was a gathering of partners within the 4M Network of Excellence, funded by the European Commission. Just one year later, the International Conference on Micro Manufacturing (ICOMM) was organised at the University of Illinois-Urbana-Champaign by the I2M2 community. In 2009, the two communities organised their first joint 4M/ICOMM conference at the Karlsruhe Institute of Technology, followed by two more joint conferences at the University of Wisconsin–Madison in 2010 and at the Politecnico di Milano in 2015. Building on the success of these jointly organised conferences, and with the support of the Asian International Forum on Micro Manufacturing, the first WCMNM was held in Taiwan, the second in Slovenia, the third in the USA, the fourth in India, and the fifth in Belgium.

This Special Issue is derived from the research presented at the fourth WCMNM held in India, where 70 research papers were presented. After peer review, the authors of 11 papers were invited to write extended manuscripts for this Special Issue of *Micromachines*. The papers consider various fields of micro and nano manufacturing, ranging from additive manufacturing, forming, and material removal processes to surface texturing and the use of artificial intelligence (AI) in process characterisation.

The parts produced, assembled, or handled using micro manufacturing technologies have at least two dimensions below 1 mm. When the size of the component is within orders of magnitude of the physical elements of the process, e.g., material grain size, the linear theory of the macro-domain breaks down. This is called the size effect [1]. Four numerical models to simulate the micro cutting of hardened steel were evaluated to choose which method of modelling can present the micro cutting process and to properly describe the size effect [2]. A comparison between different formulations led to us to conclude that the Coupled Eulerian–Lagrangian (CEL) formulation could be a promising method to model micro cutting and multi-step micro cutting in particular. However, further studies need to be carried out to optimise CEL models to simulate the micro cutting process by taking into account successive passes of the cutting edge into a machined material.

The size effect was also explored in forming processes, which is an enabler of cost-effective mass production. Micro formation at room temperature offers the potential to meet the increasing demand for metallic micro components emerging from global trends to save weight, space, materials, and costs. During micro formation, size effects occur, which negatively affect the part quality, process stability, tool life, and handling. A multi-stage bulk micro forming process from sheet metal was investigated in terms of the basic feasibility and the occurrence of size effects [3]. It was found that a strengthened material state increases the material utilisation, but the size effects cannot be eliminated by reducing the grain size. Cold forming was also explored for the manufacture of micro gears from coil material. Within the current boundaries of the technology, the cold forming of modules m < 0.2 mm is not possible due to size effects, high tool stresses, and handling problems. A novel process chain for the multi-step formation of micro gears with a module of m = 0.1 mm was proposed, and the numerical model of the first two steps of the process chain was set up and confirmed based on experimental forming tests [4]. The process chain consisted of forward extrusion, lateral extrusion, and shear cutting. No size effects regarding die filling were determined, and complete tooth filling was achieved. In addition, further tests with samples of different grain sizes should be carried out for a better understanding of the influence of size effects. The surface properties of the punch and die tool played an important role in the micro extrusion process due to size effects and frictional effects. On a billet AA6063, the effects of tool surface properties such as punch surface grooves on micro extrudability, assessed using extrusion force, and the shape of the extrusion and Vickers hardness were investigated [5]. The tribology between the tool and the material was controlled by introducing 5 µm deep grooves distributed by about 10 grooves per mm. Punches with structured surfaces exhibited better material flowability and uniformly introduced more strain.

The techniques for bearing condition monitoring are employed based on either vibration or acoustic signals, which are collected through run-to-failure or degradation tests. However, these tests are time-consuming and thus do not apply to the verification of certain detection technology, for instance, a machine-learning-based method, which requires a huge training set. For this reason, artificial defects are often induced in the form of indentation, line spalling, or square wear to shorten the incipient period of growing bearing faults. Micro EDM has been put forward as a potential technique for the fabrication of artificial defects using the drilling/milling mode [6]. A methodology has been developed not only to achieve full control of the dimension and distribution of defects on a bearing element but also to qualitatively and quantitatively perform the efficient characterisation of the defect surface.

Surface texturing is another important field for industrial applications. To harden a surface or remove surface irregularities, the cavitation peening (CP) and cavitation abrasive jet polishing (CAJP) processes can be utilised. The limitation of the zero-incidence angle of these processes was improved by introducing a secondary jet [7]. The secondary jet interacts with the main jet, carrying bubbles to the proximity of the workpiece surface and aligning the disordered bubble collapse events. In terms of characterising the treated surface of AL6061, the secondary jet increases the hardening intensity by 10%, whereas the material removal rate within a localised region increases by 66%. The hardening effect of the cavitation increases with the processing time at first and is then saturated. Additionally, multiple secondary jets can create a patched pattern of hardness distribution. Another option presented in this Special Issue is a three-step process chain consisting of plasma nitriding, femtosecond laser printing, and CNC imprinting. It was used on a punch and die to improve the design flexibility of the micro/nano textures of products, prolong the tool life, and ensure high cost-competitiveness [8]. The laser-induced periodic surface structuring (LIPSS) of the nitrided die has chemical stability and heat resistance sufficient to perform high-temperature injection moulding and mould stamping for repetitive duplication operations. Simultaneous laser trimming and nano texturing of the punch work adjusts the surface roughness and geometric irregularities by trimming and superposing the nano textures onto the trimmed surface. Such punch and die tools were used to pierce electrical steel sheets [9]. By utilising a surface-textured punch and die, the aim is to reduce the formation of plastically affected zones and thus iron losses in the core of an electric motor. The surface structure and chemistry of polymers may be altered by the incorporation of additives in the polymer structure. To enhance the polystyrene (PS) hydrophobicity, a synthesised tailored fluorinated copolymer named POISE-a (Polymer prOcessing Interface StabilizEr) was added [10], and hot embossing was used for the micro structuration of a polymer surface. The use of this tailored additive, even at low percentages (i.e., 1 wt.%), was associated with the structuration of the PS surface, and it improved both the hydrophobicity of polystyrene and the robustness of the replication process.

Additive manufacturing technologies are gaining importance in the production of micro products/parts. Drop-on-demand (DoD) inkjet printing is a high-precision, non-contact, and maskless additive manufacturing technique employed in the production of high-precision, micrometre-scaled geometries, allowing free design manufacturing for flexible devices and printed electronics. The influence of the main printing parameters (namely, the spacing between subsequent drops deposited on the substrate, the printing speed, and the nozzle temperature) on the accuracy of a representative geometry consisting of two interdigitated comb-shaped electrodes were examined [11]. A drop spacing of 140 µm and 170 µm on the two main directions of the printing plane, with a nozzle temperature of 35 °C, was shown to be the most appropriate parameter combination for use in printing the target geometry. No significant influence of the printing speed on the process outcomes was found; thus, choosing the highest speed value within the investigated range can increase productivity.

Artificial intelligence (AI) is a fast-growing digital technology which is also gaining importance in engineering. An example of AI used in bioengineering is the automatic determination of the frequency range for positive and negative dielectrophoresis (DEP)—an electrokinetic force that can be used for massively parallel micro and nano assembly [12]. The AI-guided platform has determined that positive DEP is active below 500 kHz frequency, negative DEP is evidenced above 1 MHz frequency, and the crossover frequency is between 500 kHz and 1 MHz. These results are in line with the previously published, experimentally determined, frequency-dependent DEP behaviour of latex microbeads. The phenomenological approach assisted by a live AI-guided feedback loop will assist the active manipulation of the system towards the desired phenomenological outcome such as, for example, the collection of particles at electrodes, even if, due to the complexity and plurality of the interactive forces, model-based predictions are not available.

Finally, we thank all of the authors for their excellent research, as well as the reviewers for their time and efforts devoted to the peer-review processes, which were essential to improve the quality of the manuscripts. Last but not least, we thank the editorial staff of *Micromachines* for their full support in preparing this Special Issue.

## References

[1] Qin Y. (2015). Micromanufacturing Engineering and Technology.

[2] Chaabani L., Piquard R., Abnay R., Fontaine M., Gilbin A., Picart P., Thibaud S., D’Acunto A., Dudzinski D. (2022). Study of the Influence of Cutting Edge on Micro Cutting of Hardened Steel Using FE and SPH Modeling. Micromachines.

[3] Kraus M., Merklein M. (2021). Investigation of Size Effects in Multi-Stage Cold Forming of Metallic Micro Parts from Sheet Metal. Micromachines.

[4] Rohrmoser A., Kraus M., Merklein M. (2021). Forming of Components with Mi-crogearings from Coil Material—Numerical Modeling of the Process Chain and Experimental Validation. Micromachines.

[5] Funazuka T., Dohda K., Shiratori T., Hiramiya R., Watanabe I. (2021). Effect of Punch Surface Grooves on Microformability of AA6063 Backward Microextrusion. Micromachines.

[6] Ye L., Saxena K.K., Qian J., Reynaerts D. (2022). Micro-EDM Drilling/Milling as a Potential Technique for Fabrication of Bespoke Artificial Defects on Bearing Raceways. Micromachines.

[7] Pang H., Ngaile G. (2022). Utilization of Secondary Jet in Cavitation Peening and Cavitation Abrasive Jet Polishing. Micromachines.

[8] Aizawa T., Yoshino T., Suzuki Y., Inohara T. (2022). Micro-/Nano-Texturing onto Plasma-Nitrided Tool Surface by Laser Printing for CNC Imprinting and Piercing. Micromachines.

[9] Aizawa T., Shiratori T., Yoshino T., Suzuki Y., Dohda K. (2022). Quantitative Characterization of the Affected Zones in a Single Crystal Fe-6Si Steel Sheet by Fine Piercing. Micromachines.

[10] le Brouster R., Giboz J., Nourdine A., Tenchine L., Dubelley F., Mele P. (2022). Complementary Approaches for Enhancing Polystyrene Hydrophobicity: Additives Development and Replication of Micro/Nanotextures. Micromachines.

[11] Bertolucci F., Berdozzi N., Rebaioli L., Patil T., Vertechy R., Fassi I. (2021). Assessing the Relationships between Interdigital Geometry Quality and Inkjet Printing Parameters. Micromachines.

[12] Michaels M., Yu S.-Y., Zhou T., Du F., al Faruque M.A., Kulinsky L. (2022). Artificial Intelligence Algorithms Enable Automated Characterization of the Positive and Negative Dielectrophoretic Ranges of Applied Frequency. Micromachines.

